# Mining of Cyanobacterial Genomes Indicates Natural Product Biosynthetic Gene Clusters Located in Conjugative Plasmids

**DOI:** 10.3389/fmicb.2021.684565

**Published:** 2021-11-04

**Authors:** Rafael Vicentini Popin, Danillo Oliveira Alvarenga, Raquel Castelo-Branco, David Peter Fewer, Kaarina Sivonen

**Affiliations:** ^1^Department of Microbiology, University of Helsinki, Helsinki, Finland; ^2^Department of Biology, University of Copenhagen, Copenhagen, Denmark; ^3^Interdisciplinary Centre of Marine and Environmental Research (CIIMAR), University of Porto, Porto, Portugal

**Keywords:** cyanobacteria, conjugation, genome mining, toxins, geosmin

## Abstract

Microbial natural products are compounds with unique chemical structures and diverse biological activities. Cyanobacteria commonly possess a wide range of biosynthetic gene clusters (BGCs) to produce natural products. Although natural product BGCs have been found in almost all cyanobacterial genomes, little attention has been given in cyanobacterial research to the partitioning of these biosynthetic pathways in chromosomes and plasmids. Cyanobacterial plasmids are believed to disperse several natural product BGCs, such as toxins, by plasmids through horizontal gene transfer. Therefore, plasmids may confer the ability to produce toxins and may play a role in the evolution of diverse natural product BGCs from cyanobacteria. Here, we performed an analysis of the distribution of natural product BGCs in 185 genomes and mapped the presence of genes involved in the conjugation in plasmids. The 185 analyzed genomes revealed 1817 natural products BGCs. Individual genomes contained 1–42 biosynthetic pathways (mean 8), 95% of which were present in chromosomes and the remaining 5% in plasmids. Of the 424 analyzed cyanobacterial plasmids, 12% contained homologs of genes involved in conjugation and natural product biosynthetic pathways. Among the biosynthetic pathways in plasmids, manual curation identified those to produce aeruginosin, anabaenopeptin, ambiguine, cryptophycin, hassallidin, geosmin, and microcystin. These compounds are known toxins, protease inhibitors, odorous compounds, antimicrobials, and antitumorals. The present study provides *in silico* evidence using genome mining that plasmids may be involved in the distribution of natural product BGCs in cyanobacteria. Consequently, cyanobacterial plasmids have importance in the context of biotechnology, water management, and public health risk assessment. Future research should explore *in vivo* conjugation and the end products of natural product BGCs in plasmids via chemical analyses.

## Introduction

Microbial natural products originate from secondary metabolism and exhibit a wide range of chemical structures and biological activities (Woodruff, 1980). These metabolites can act as antibiotics, anticancer agents, antivirals, and toxins and can be used as enzyme inhibitors, polymers, or surfactants (Demain, 2014). The enzymes involved in the biosynthesis of natural products are commonly encoded in biosynthetic gene clusters (BGCs) located in contiguous stretches of DNA (Stone and Williams, 1992; Osbourn, 2010; Jensen, 2016). Natural product BGCs usually include core biosynthesis, regulatory and resistance, and tailoring genes (Daum et al., 2009; Nett et al., 2009). Among accessory enzymes, 4-phosphopantetheinyl transferases (PPTs) play a major role in the biosynthesis of several types of natural products (Lambalot et al., 1996; Beld et al., 2014; Yang et al., 2017).

Understanding of the genetic diversity and distribution of natural product BGCs has greatly increased in the last decade due to the enormous expansion in the number of sequenced bacterial genomes in the last decade (Land et al., 2015; Jensen, 2016). Genome mining, which uses bioinformatics techniques to identify genes encoding enzymes possibly involved in natural products biosynthesis, has led to the discovery of novel compounds (Corre and Challis, 2009; Zerikly and Challis, 2009; Bachmann et al., 2014). Cyanobacteria are among several phyla of bacteria that are commonly explored using these techniques (Micallef et al., 2015).

Since the genome of *Synechocystis* sp. PCC 6803 was sequenced in 1996 (Kaneko et al., 1996), the number of complete cyanobacterial genomes deposited in the NCBI GenBank has slowly increased in comparison to other bacteria (Alvarenga et al., 2017). Despite their underrepresentation in public databases, cyanobacterial genomes were successfully investigated from evolutionary, ecological, and taxonomic perspectives (Sciuto and Moro, 2015). Cyanobacteria are recognized as a source of diverse natural products with applications in pharmacology, biotechnology, and bioenergy production (Burja et al., 2001; Angermayr et al., 2009; Kehr et al., 2011). A considerable portion of these molecules is produced by non-ribosomal peptide synthetases (NRPSs) and polyketide synthases (PKSs) (Tan, 2007; Dittmann et al., 2015). Other classes of broadly distributed cyanobacterial natural products include ribosomally synthesized and post-translationally modified peptides (RiPPs), alkaloids, and terpenoids, among others (Hillwig et al., 2014; Martins and Vasconcelos, 2015; Pattanaik and Lindberg, 2015; Jain et al., 2017). Genome mining of cyanobacterial genomes has helped unravel the diversity of BGCs involved in the production of various natural products (Singh et al., 2010; Wang et al., 2011; Micallef et al., 2015).

Cyanobacterial natural product BGCs are mostly concentrated in the genomes of late-branching cyanobacteria, mainly in the orders Oscillatoriales and Nostocales, although they are found in almost all cyanobacterial genomes (Shih et al., 2013; Calteau et al., 2014; Dittmann et al., 2015). Several studies have mapped the distribution of BGCs in these organisms (Dittmann et al., 2015). However, in cyanobacterial studies, little attention has been given to BGCs located in plasmids (Ehrenreich et al., 2005; Wang et al., 2011; Shih et al., 2013; Calteau et al., 2014; Dittmann et al., 2015). Nevertheless, horizontal gene transfer events are linked to the dissemination and evolution of many cyanobacterial natural product BGCs, including toxins such as cylindrospermopsin, microcystin, anatoxin-a, and saxitoxin (Lawrence and Roth, 1996; Ginolhac et al., 2005; Dittmann et al., 2013). This hypothesis places importance on cyanobacterial plasmids, as they would be directly involved in differentiating toxic and non-toxic strains, and in production of several natural products with economic, environmental, and public health importance (Tooming-Klunderud et al., 2008; Dittmann et al., 2013).

Plasmids play a key role in horizontal gene transfer, and conjugation is one of the processes that can transfer genetic material (Boto, 2010; Harrison and Brockhurst, 2012). The most frequent mechanism of DNA conjugation in gram-negative bacteria involves a relaxome, which includes a relaxase and a type IV coupling protein (T4CP) encoded by mobility genes (MOB), and a transferosome assembled by a type IV secretion system (T4SS) that is encoded by mating pair formation genes (MPF) (De La Cruz et al., 2010; Smillie et al., 2010). During conjugation, the relaxase cleaves and covalently binds itself to the transferring DNA on a site called *oriT* (Garcillán-Barcia et al., 2009). The T4SS is believed to then act as a secretor protein by transferring DNA and the relaxase to the recipient cell (Christie, 2004). For this purpose, the T4CP recognizes, energizes, and delivers the nucleoprotein to the T4SS (Zechner et al., 2012). Plasmids encoding these three components are called self−transmissible or conjugative, while mobilizable plasmids usually encode just the MOB and a T4SS and are transmitted only in the presence of a helper conjugative plasmid (Garcillán-Barcia et al., 2009). Most cyanobacterial plasmids were predicted to lack the necessary genes to be conjugative (Smillie et al., 2010). However, no concomitant analysis of the presence of BGCs in plasmids and the mobility of these replicons is currently available for cyanobacteria.

Thus, the present study screened 184 complete genomes publicly available in the GenBank database (Clark et al., 2016) from the phylum Cyanobacteria and one from *Candidatus* Melainabacteria, a phylum that is closely related to cyanobacteria (Di Rienzi et al., 2013). We used genome mining to provide evidence that several known natural product BGCs are in plasmids, some of which might be conjugative.

## Materials and Methods

### Cyanobacterial Genomes

“Cyanobacteria/Melainabacteria group” genomes deposited until January 14, 2020 in the NCBI GenBank (Clark et al., 2016) at “Complete” and “Chromosome” assembly levels were analyzed. Altogether, they included 184 genomes from the phylum Cyanobacteria and 1 genome from the closely related *Candidatus* Melainabacteria (Supplementary Table 1; Di Rienzi et al., 2013). Statistics of the genome assemblies were obtained from the NCBI GenBank website (Clark et al., 2016). Means, *p*-value, averages, standard deviations, and boxplot and scatter graphs were generated using standard analyses in Microsoft Excel v16.0.6742.2048 (Microsoft, Redmond, WA). Pearson and Spearman’s rank correlation coefficients were calculated in R Project for Statistical Computing version 4.0.5.^1^

### Identification of Natural Product Pathways and Other Proteins of Interest

BGCs involved in natural product biosynthetic pathways were automatically annotated using the “Bacterial” sequence analysis in antiSMASH v5.1.1 (Blin et al., 2019). For this analysis, the standard “relaxed” strictness was applied, and no extra future was activated. Manual annotation and curation of BGCs were performed in the program Artemis v18.1.0 (Carver et al., 2012). This manual step involved comparisons of automatically annotated BGCs to diverse previously described reference gene clusters from cyanobacterial strains using the protein searches with BLASTp (default parameters were used; see Supplementary Table 1 for alignment scores; Altschul et al., 1990). Searches for PPTs were performed using BLASTp searches of candidate proteins in the analyzed genomes (default parameters; Altschul et al., 1990) using the 4’-phosphopantetheinyl transferase from *Nostoc* sp. PCC 7120 (NCBI accession number P37695.2) as reference. The best identified possible homologs (proteins with the highest *E*-value) were compared using BLASTp (default parameters; Altschul et al., 1990) to the previously described 4’-phosphopantetheinyl transferase type Sfp from *Bacillus cereus* (WP_002185911.1) and holo-acyl carrier protein synthase (type A, accession number NP_388343.1) from *Bacillus subtilis* subsp. *subtilis* str. 168 (see Supplementary Table 2 for search scores). Identification of relaxase (accession number in NCBI: MBD2275468.1), VirB4 (BAB78290.1), and VirD4 (MBD2275482.1) were performed with BLASTp protein searches (default parameters; Altschul et al., 1990) using genes of the known conjugative plasmid pCC7120α from *Nostoc* sp. PCC 7120 (see Supplementary Table 3 for scores of manually curated best candidate genes in plasmids with known BGCs and Supplementary Table 4 for automatic searches for candidate genes in the other plasmids using cutoff of coverage 80%, identity 30%, and *E*-value 1.00 E-20).

Plasmid representations were generated using the standard parameters of the “BLAST analysis” in the server Gview (Petkau et al., 2010) and default analysis in BRIG v0.95 (Alikhan et al., 2011). The program Inkscape v0.92 was used for drawing BGCs.^2^

### Phylogenetic Analyses

The phylogenetic analyses of the concatenated amino acid sequences from the BGCs and nucleotide sequences of 16S rRNA were created with 5,000,000 generations in MrBayes 3.2.7a (Ronquist et al., 2012). The best substitution model for each protein in the BGCs was predicted using the default BIC calculation in ProtTest 3.4.2 (Darriba et al., 2011). Default BIC calculation was also used in jModelTest v2.1.10 (Darriba et al., 2012) for the 16S rRNA phylogenetic analyses; and the model HKY + I + G was predicted as the best. Tree visualization was performed with FigTree v1.4.4^3^ and editing with Inkscape 1.0 (see text footnote 2). Cyanobacterial order was assigned according to a polyphasic taxonomic system (Komarek et al., 2014).

### Phylogenomic Analysis

A maximum likelihood tree was constructed in RAxML v8.2.12 (Stamatakis, 2014) using 1,000 bootstrap samples. The model LG + G + I was identified as the best-fitting model by default BIC calculation in ProtTest 3.4.2 (Darriba et al., 2011). The phylogenomic placement was based on concatenated amino acid sequences of 120 bacterial single-copy conserved marker genes identified by GTDBTk 1.0.2 (default parameters; Parks et al., 2018). The tree was visualized in FigTree v1.4.4 (see text footnote 3) and figure editing was performed in Inkscape 1.0 (see text footnote 2).

## Results

**FIGURE 1:**
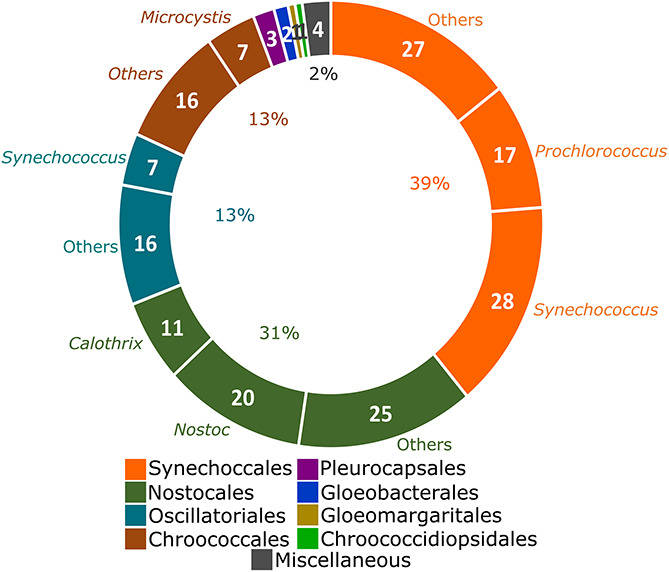
Taxonomic distribution of the 184 complete genomes deposited in the NCBI Genbank (Clark et al., 2016) from Cyanobacteria (Komarek et al., 2014) and one from the closely related phylum *Candidatus* Melainabacteria (Di Rienzi et al., 2013). Strains that remain unclassified in any cyanobacterial order and the *Candidatus* Melainabacteria strain MEL.A1 were allocated as Miscellaneous. *Synechococcus* is a polyphyletic genus (Komarek et al., 2020). Genera over-represented in each order are indicated. The number of genomes from over-represented genera is presented and the percentages were calculated in relationship to the total of 185 genomes.

The taxonomic distribution of analyzed genomes was initially explored. Following the latest proposed system of cyanobacteria, 39% of the analyzed genomes belong to the order Synechococcales (mainly *Synechococcus*, a polyphyletic genus with representatives also allocated in Oscillatoriales and *Prochlorococcus*), followed by Nostocales (31% of genomes; largely represented by *Nostoc* and *Calothrix*) (Figure 1). The remaining 30% of the genomes were distributed in the orders Oscillatoriales, Chroococcales, Pleurocapsales, Gloeobacterales, Gloeomargaritales, and Chroococcidiopsidales (Figure 1). No representative genome of the order Spirulinales was analyzed here due to unavailability. The Miscellaneous category includes three unclassified cyanobacterial genomes and the *Candidatus* Melainabacteria strain MEL.A1.

### General Features of the Evaluated Genomes

From the 52 genera represented in the retrieved dataset, 27 included more than one genome. For these genera, basic statistical analyses (averages and standard deviation) were used and revealed insights into the genomic differences of cyanobacterial orders (Table 1). According to the NCBI GenBank database, these cyanobacterial genomes consisted of up to two chromosomes (calculated median of 1 per genome) and 14 plasmids (median of 1; Supplementary Table 5). Chromosomes represented from 97 to 100% of the strain’s genomic DNA, while plasmids represented up to 3% (Supplementary Table 5). Genome sizes ranged from 1.65 to 12.05 Mb (median 5.02 Mb), GC content from 30.8 to 68.7% (median of 42.2%), and the number of genes varied from 1816 to 11674 (median 4553) (Supplementary Table 5). The number of BGCs automatically annotated by antiSMASH v5.1.1 in chromosomes ranged from 1 to 42 (mean 8); up to five were found located in plasmids (median 0).

**TABLE 1:** Genome statistics of cyanobacterial genera with more than one complete sequence in the NCBI GenBank (Clark et al., 2016).

**Order**	**Genus**	**No. gen**	**Pld/gen**	**Size (Mb)**	**GC (%)**	**Genes**	**No. BGCs Chr**	**No. BGCs Pld**	**Total BGCs**
Gloeobacterales	*Gloeobacter*	2	0	4.69 ± 0.04	61.3 ± 1.1	4,497 ± 21	5 ± 3	0	7 ± 1
Synechococcales	*Cyanobium*	2	0	3.18 ± 0.23	68.7 ± 0.1	3,227 ± 251	8 ± 1	0	8 ± 1
	*Leptolyngbya*	6	2 ± 2	6.37 ± 0.85	47.9 ± 4.1	5,890 ± 965	10 ± 4	1 ± 1	11 ± 5
	*Prochlorococcus*	17	1 ± 0	1.83 ± 0.29	34.8 ± 6.3	2,018 ± 327	6 ± 5	1 ± 0	6 ± 5
	*Pseudanabaena*	2	4 ± 4	5.28 ± 0.54	44.2 ± 2.8	4,510 ± 789	3 ± 1	1 ± 0	4 ± 3
	*Synechococcus*	28	0 ± 1	2.79 ± 0.57	57.2 ± 5.4	2,847 ± 452	6 ± 4	0	5 ± 4
	*Synechocystis*	8	2 ± 3	3.72 ± 0.18	47.6 ± 0.2	3,465 ± 200	3 ± 0	0	3 ± 0
	*Thermosynechococcus*	5	0	2.55 ± 0.06	53.7 ± 0.3	2,543 ± 67	3 ± 0	0	3 ± 0
Oscillatoriales	*Arthrospira*	3	0	6.47 ± 0.35	44.4 ± 0.3	8,118 ± 3,081	3 ± 1	0	3 ± 1
	*Moorea*	2	2 ± 1	9.55 ± 0.23	43.6 ± 0.1	7,748 ± 31	42 ± 0	0	42 ± 0
	*Oscillatoria*	2	4 ± 2	8.04 ± 0.33	46.7 ± 1.3	6,479 ± 590	8 ± 1	1 ± 0	9 ± 2
	*Planktothrix*	2	5 ± 1	5.07 ± 0.02	39.6 ± 0.1	4,538 ± 13	6 ± 4	2 ± 0	11 ± 1
	*Synechococcus*	7	5 ± 1	3.14 ± 0.12	49.2 ± 0.1	3,183 ± 117	3 ± 0	1 ± 1	4 ± 1
Chroococcales	*Cyanobacterium*	2	1 ± 1	3.23 ± 0.10	38.2 ± 0.7	3,020 ± 251	4 ± 1	0	4 ± 1
	*Gloeothece*	2	6 ± 0	7.20 ± 0.91	39.2 ± 1	6,406 ± 795	8 ± 1	5 ± 4	13 ± 2
	*Geminocystis*	3	8 ± 4	4.24 ± 0.20	33.3 ± 1.0	3,831 ± 270	4 ± 1	0	4 ± 1
	*Microcystis*	7	0	5.19 ± 0.65	42.5 ± 0.3	5,243 ± 752	10 ± 1	0	10 ± 1
	*Rippkaea*	2	4 ± 1	4.80 ± 0.01	39.8 ± 0.0	4,540 ± 30	8 ± 0	0	8 ± 0
Pleurocapsales	*Stanieria*	2	3 ± 3	5.50 ± 0.06	36.4 ± 0.2	4,948 ± 30	11 ± 2	1 ± 0	12 ± 3
Nostocales	*Anabaena*	5	3 ± 3	6.44 ± 0.83	39.1 ± 1.3	5,713 ± 558	14 ± 3	1 ± 1	14 ± 3
	*Calothrix*	11	4 ± 3	8.70 ± 2.11	40.2 ± 1.5	7,273 ± 1,957	15 ± 6	1 ± 0	15 ± 6
	*Cylindrospermum*	2	4 ± 1	7.66 ± 0.06	42.1 ± ± 0.1	6,574 ± 65	21 ± 3	3 ± 2	24 ± 1
	*Dolichospermum*	2	2 ± 2	5.41 ± 0.33	38.2 ± 0.0	5,032 ± 349	11 ± 4	0	11 ± 4
	*Fischerella*	3	5 ± 4	6.54 ± 1.00	40.5 ± 0.7	5,547 ± 899	13 ± 1	5 ± 0	15 ± 3
	*Nodularia*	2	1 ± 1	5.43 ± 0.05	41.2 ± 0	4,866 ± 42	11 ± 1	0	11 ± 1
	*Nostoc*	20	5 ± 3	7.88 ± 1.22	41 ± 0.8	6,824 ± 1,096	16 ± 5	2 ± 2	18 ± 6
	*Scytonema*	2	6 ± 2	9.81 ± 0.06	43.6 ± 0.2	8,176 ± 76	25 ± 1	2 ± 1	27 ± 2
	*Trichormus*	2	4 ± 1	7.29 ± 0.25	41.4 ± 0	6,147 ± 291	14 ± 1	2 ± 1	15 ± 1

*Averages and standard deviations of genome size, GC content, number of genes, BGCs in the chromosomes and plasmids, and the total number of BGCs in the genome were calculated.*

*Gen, genome; Pld, plasmid; BGC, biosynthetic gene cluster; Chr, chromosome.*

### Overall Biosynthetic Potential

**FIGURE 2:**
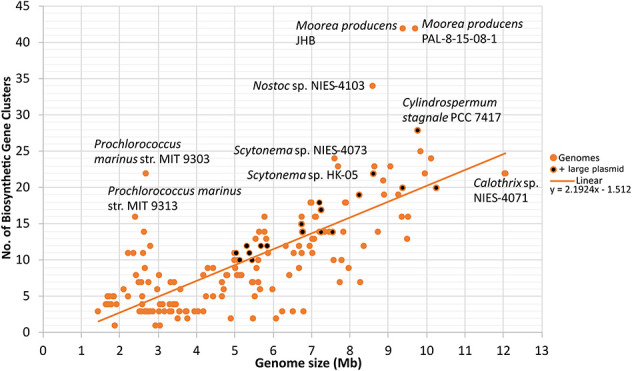
Number of automatically predicted natural product biosynthetic gene clusters plotted against genome size (Mb). Genomes with large plasmids (>500 kb) are also indicated (diCenzo and Finan, 2017). Linear trend and its corresponding linear correlation coefficient are presented. *N* = 185.

The analyzed cyanobacterial genomes were automatically annotated and a total of 1817 BGCs were identified (Supplementary Table 5). *Synechococcus* sp. JA-2-3B’a(2–13), *Candidatus* Melainabacteria MEL.A1, and *Synechococcus* sp. JA-3-3Ab had only one natural product BGC and thus were the genomes with the lowest number. In contrast, *Moorea producens* JHB and *Moorea producens* PAL-8-15-08-1 had 42 BGCs each (Supplementary Figure 1). Nostocales genomes were among those with the highest average number of natural product BGCs (Supplementary Figure 1). A positive correlation between the number of natural product BGCs and genome size was found (Pearson *r* = 0.75, *p* < 0.001; Spearman’s rho = 0.71, *p* < 0.001; Figure 2).

When the distribution of the automatically annotated BGCs in specific replicons was considered, most BGCs were identified in chromosomes (1718) and represented 95% of the total (1817). RiPPs were the most widespread class of natural product BGC products in these replicons (526 representatives, 31% of the chromosomal BGCs). Terpenes were the second most widespread products (470 representatives, 27% of the natural product BGCs in chromosomes) and were absent only in *Arthrospira platensis* C1, *Candidatus* Melainabacteria bacterium MEL.A1, and *Nostoc sphaeroides* CCNUC1. PKS genes were most frequently found associated with other classes, whereas only 49 single class PKS BGCs representatives were found.

A total of 424 plasmids were identified from the 185 genomes, of which 73 (17% of the total number of plasmids) had at least one natural product BGC (Supplementary Table 5). Ninety-nine BGCs were found in cyanobacterial plasmids. While most plasmids included only one natural product BGC, *Gloeothece verrucosa* PCC 7822 Cy782201 (0.88 Mb) had five (Supplementary Figure 2). Hybrid NRPS/PKS corresponded to more than half of the natural product BGCs located on plasmids (26 BGCs). NRPS consisted of 20 representatives, followed by bacteriocin (15 representatives). In contrast to chromosomes, terpenes were one of the least frequently observed products with biosynthetic pathways found on plasmids. The natural product BGCs identified as known biosynthetic pathways were further explored by manual curation.

### Manual Curation of Biosynthetic Pathways on Plasmids

Following the automatic annotation, BGCs assigned to known biosynthetic pathways were manually curated to identify false positives. Interestingly, several of the known natural product BGCs were in large replicons that were nevertheless named in the database as plasmids (here > 500 kb, reaching a maximum of about 1.78 Mb in *Nostoc linckia* NIES-25 plasmid1, Supplementary Table 5). These large replicons contained 1–5 natural product BGCs. The pathways for the known natural products ambiguine (in *Fischerella* sp. NIES-4106 plasmid 2, 550 kb), anabaenopeptin (*Scytonema* sp. HK-05 plasmid 1, 831 kb), geosmin (*Nostoc linckia* NIES-25 plasmid1, 1.78 Mb), and microcystin (*Fischerella* sp. NIES-4106 plasmid1, 550 kb) were encoded in large plasmids (Supplementary Figure 3 and Supplementary Table 5).

Other known natural product BGCs were located in smaller plasmids: i.e., aeruginosin in *Cylindrospermum* sp. NIES-4074 plasmid1 (340 kb); anabaenopeptin in *Gloeothece citriformis* PCC 7424 plasmid pP742401 (328 kb) and *Gloeothece verrucose* PCC 7822 plasmid Cy782201 (879 kb), cryptophycin in *Nostoc* sp. ATCC 53789 plasmid pNSP_c (219 kb), geosmin in *Nostoc* sp. NIES-2111 plasmid2 (320 kb), and hassallidin in *Aulosira laxa* NIES-50 plasmid1 (292 kb) and *Tolypothrix tenuis* PCC 7101 plasmid1 (292 kb) (Supplementary Table 5). Although the core enzymes for biosynthesis were found in these natural product BGCs in small plasmids, some of them are missing accessory genes that are present in the references and may produce compounds with distinct structures (Supplementary Table 1). To provide further evidence that the known natural product BGCs in plasmids are likely functional, the presence of key enzyme PPTs in the genomes was investigated.

### Distribution of 4-Phosphopantetheinyl Transferases

A total of 165 possible homologs of PPTs were found (Supplementary Table 3). From the 185 genomes analyzed here, 157 had at least one copy of PPT homologs (84% of the total number of genomes). Most of these genomes encoded only 1 enzyme (149 genomes), while 6 genomic sequences encoded 2 enzymes and 1 genome (*Halomicronema hongdechloris* C2206) had 3 copies of PPTs. The size of these enzymes ranged from 137 (one of the two copies located in *Chroococcidiopsis thermalis* PCC 7203) to 339 aa (one of the three copies in *Halomicronema hongdechloris* C2206). However, 90% of the enzymes were between 200 and 280 aa (147 PPTs).

Interestingly, *Acaryochloris marina* MBIC11017 had a chromosomal PPT that was more like an Sfp-type PPT, and another in plasmid pREB1 that was the only one predicted as an AcpS-type PPT. The remaining 163 PPTs in the analyzed cyanobacterial genomes were likely Sfp-type.

### Homologs of Proteins Involved in Conjugation

**FIGURE 3:**
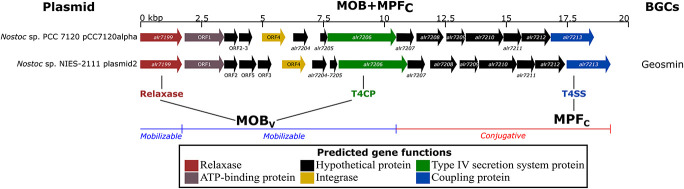
Genomic region of *Nostoc* sp. PCC 7120 plasmid pCC7120alpha and *Nostoc* sp. NIES-2111 plasmid2 encoding homologs of the main protein involved in the plasmid conjugative apparatus [relaxase- *alr7199*, *virB4*- *alr7206* (T4CP) and *virD4*- *alr7213* (T4SS)]. Previous studies characterized the mobility of plasmids (Smillie et al., 2010), MOB_v_ (Garcillán-Barcia et al., 2009), and MPF_c_ (Guglielmini et al., 2011). See Supplementary Table 4 for annotation of the relaxase, T4CP, and T4SS proteins in the 185 genomes involved in the present work.

To investigate the hypothesis of plasmids carrying natural product BGCs through conjugation, the 424 plasmids from the analyzed genomes were searched for the presence of homologs of relaxases, VirB4 (alr7206, a coupling protein, T4CP), and VirD4 (alr7213, a secretion system protein, T4SS). The reference plasmid pCC7120alpha from *Nostoc* sp. PCC 7120 harbored a similar gene cluster as that found in plasmid2 in *Nostoc* sp. NIES-2111 (Figure 3). This latter replicon was the sole plasmid encoding homolog proteins of a known natural product BGC (geosmin) to be predicted as conjugative. The results from the manual curation of known gene clusters in 10 other plasmids can be found in Supplementary Table 3.

Automatic annotation was used to predict the mobility of the remaining 414 plasmids (Supplementary Table 4). In total, 23 plasmids from Nostocales (genera *Calothrix, Cylindrospermum, Fischerella*, *Nodularia, Nostoc*, *Trichormus*) and 1 from Oscillatoriales (*Microcoleus*) contained relaxases, VirB4, and VirD4; and were predicted as conjugative. This represents 6% of the 424 analyzed cyanobacterial plasmids. Another plasmid from Chroococcales (*Gloeocapsa*), 24 from Nostocales (*Anabaena, Aulosira*, *Calothrix, Nostoc, Trichormus, Tolypothrix*), and 1 from Oscillatoriales (*Crinalium*) were predicated to be mobilizable as they encoded the relaxase but not Virb4 and VirD4. They also harbored 6% of the 424 evaluated cyanobacterial plasmids. The remaining 374 (88%) plasmids had only Virb4 or VirD4 and are likely immobile according to the current model.

Therefore, our results indicated that 15 possibly conjugative plasmids harbored a total of 18 natural product BGCs (including the known biosynthetic pathways for geosmin) and 4 other mobilizable plasmids with 1 natural product BGC each.

Phylogenetic trees were built with the manually curated known natural product BGCs found both in plasmids and chromosomes to investigate divergent evolutionary history (Supplementary Figures 4B–E). Phylogenetic trees based on hassallidin, geosmin, and anabaenopeptin biosynthetic enzymes showed that BGCs from plasmids appear to be more closely related than the chromosomal natural product BGCs. Thus, natural product BGCs from plasmids possibly face different evolutionary pressures than those present in chromosomes. A single microcystin BGC was found in plasmids included in the present study and formed a clade with a BGC from a chromosome (Supplementary Figure 4D).

### Taxonomic Distribution of Natural Product Biosynthetic Gene Clusters, Phosphopantetheinyl Transferases, and Mobile Plasmids

**FIGURE 4:**
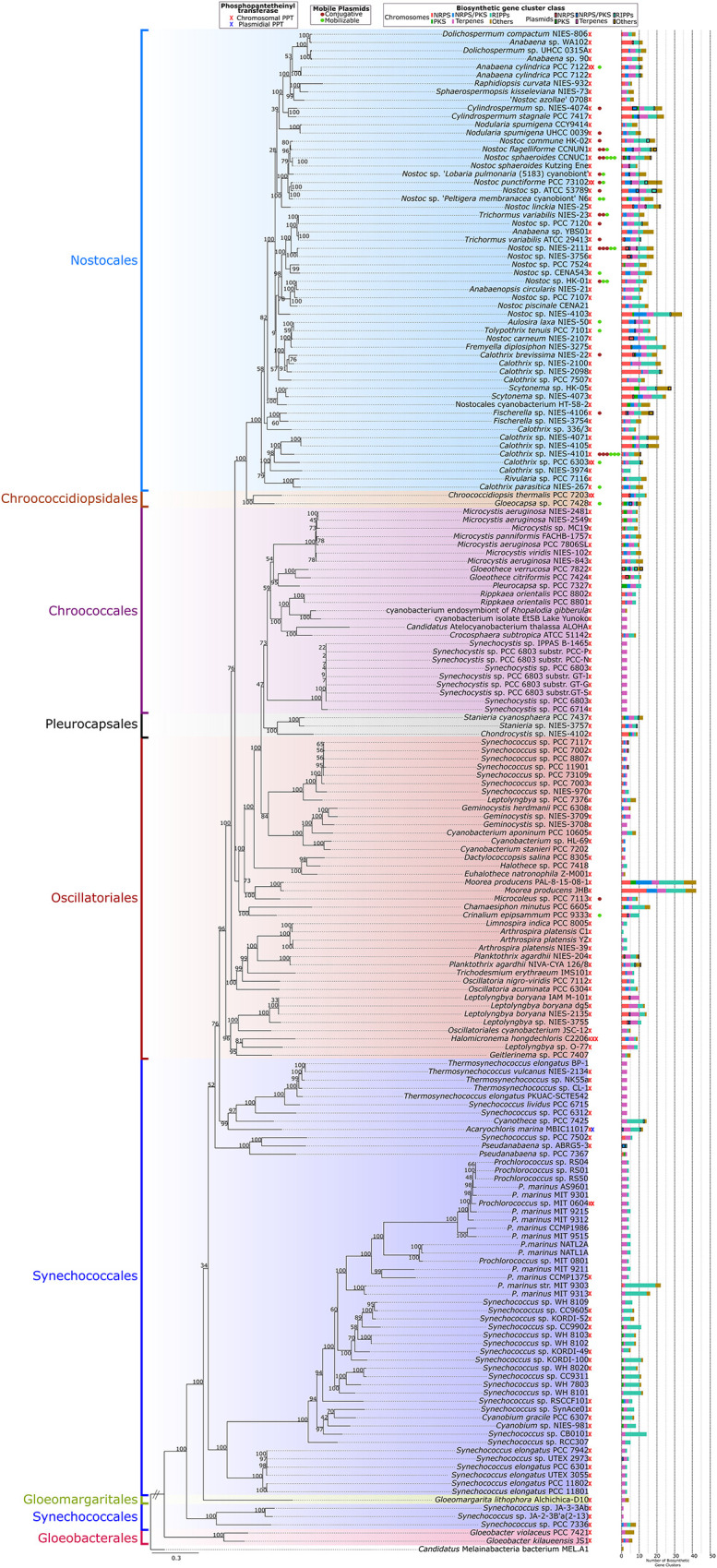
Phylogenomic tree using 120 conserved proteins from the 185 complete genomes in this study using *Candidatus* Melainabacteria bacterium MEL.A1 as outgroup. The number of phosphopantetheinyl transferases, mobilizable and conjugative plasmids, and biosynthetic gene clusters in the genomes are presented for each strain (*x*-axis).

A phylogenomic tree was used to map the taxonomical distribution of natural product BGCs, PPTs, and mobile plasmids (conjugative and mobilizable) in the analyzed cyanobacterial genomes (Figure 4). Natural product BGCs and PPTs were detected in all the cyanobacterial orders included in the present study. However, later-branching cyanobacteria from Nostocales had most of the natural product BGCs and a clear majority of the mobile plasmids. In contrast, early branching cyanobacteria, especially those from Gloeobacterales, Synechococcales, and Gloeomargaritales, had fewer natural product BGCs (mainly terpenes and RiPPs) and no mobile plasmids. A supplementary 16S rRNA phylogenetic tree of the analyzed genomes is shown in Supplementary Figure 4A.

## Discussion

Based on the model Gram-negative bacteria *Escherichia coli*, cyanobacteria are commonly assumed to be monoploid (Griese et al., 2011). However, these organisms can become oligoploid during rapid growth (Skarstad et al., 1983; Blank and Sánchez-Baracaldo, 2010). Some cyanobacteria may contain multiple chromosome copies throughout their life cycles (Griese et al., 2011; Sargent et al., 2016; Watanabe, 2020). Previous studies reported polyploidy in several genera, such as *Anabaena* (*Nostoc)* (Hu et al., 2007), *Synechococcus* (Chen et al., 2012), and *Synechocystis* (Schneider et al., 2007). Here, *Nostoc sphaeroides* CCNUC1, *Crocosphaera subtropica* ATCC 51142 (Welsh et al., 2008), and *Anabaena (Dolichospermum)* sp. 90 (Wang et al., 2012) were found to possess more than one chromosome. In contrast, large plasmids (considered here as > 500 kb) were found in 11 genomes from 4 of the 6 analyzed cyanobacterial orders and could correspond to previously described “chromids” (diCenzo et al., 2019).

Chromids are large, plasmid-like replicons that were previously found in approximately 10% of bacterial genomes (Harrison et al., 2010). Chromids possess replication systems that are similar to plasmids and can carry essential genes for cell viability (diCenzo and Finan, 2017). One of the proposed functions of these large replicons is to increase genome plasticity through the rapid acquisition or loss of genes by horizontal gene transfer (diCenzo et al., 2019). Chromids occurred in approximately 15% of the analyzed cyanobacterial genomes and contained several natural product BGCs automatically annotated.

Tools for automatic annotation of natural product BGCs were initially proposed to be a solution to the labor-intensive and time-consuming task of manual curation and, currently, antiSMASH is widely used in cyanobacterial research (Medema et al., 2011; Calteau et al., 2014; Micallef et al., 2015; Leao et al., 2017). The algorithm behind the identification of the biosynthetic pathways has been constantly updated to improve detection and prevent false positives (Weber et al., 2015; Blin et al., 2017, 2019). For instance, more pathways for the biosynthesis of RiPPs and PKS BGCs were detected using the latest versions of the tool compared with initial versions (Weber et al., 2015; Blin et al., 2017). Therefore, the fact that the analyzed genomes contained on average eight natural product BGCs in comparison to the previous estimation of five, should be approached with caution (Dittmann et al., 2015). Here, most of the automatically annotated natural products were predicted to have RiPPs and terpenes as end products.

Natural product BGCs encoding enzymes for RiPPs were found in almost all analyzed genomes. These molecules are products of post-translational modification of ribosomally synthesized precursor peptides (Arnison et al., 2013). Thus far, over 20 families of compounds that possess unique chemical features have been proposed (Arnison et al., 2013). Cyanobacteria encode the machinery to produce several RiPPs, including cyanobactins (Sivonen et al., 2010), lanthipeptides (Cubillos-Ruiz et al., 2017), lasso peptides (Tietz et al., 2017), and microviridins (Ziemert et al., 2010). Considering that automated tools are being improved to better predict genes involved in the biosynthesis of these compounds, future studies may expand the known repertoire of RiPPs produced by cyanobacteria (Blin et al., 2017, 2019).

Like RiPPs, genes for the biosynthesis of terpenes are widespread in cyanobacterial genomes (Shih et al., 2013). Although terpenes are commonly isolated from plants and fungi, terpene BGCs are widely found in bacterial genomes (Yamada et al., 2015). These compounds are essential in primary metabolism, such as for photosynthesis and respiration, but also have roles as secondary metabolites (Breitmaier, 2006). These roles likely explain why genes encoding enzymes involved in the biosynthesis of terpenes are present in cyanobacterial genomes (Leao et al., 2017). The repertoire of terpenes produced by cyanobacteria is possibly larger than currently known, as various cryptic terpene synthases are commonly found in their genomes (Shih et al., 2013; Yamada et al., 2015). Thus far, geosmin and 2-methylisoborneol are widely studied terpenes as they are odorous metabolites that impact drinking water quality (Jüttner, 1995; Suurnäkki et al., 2015; Oh et al., 2017). A pathway for geosmin production was among the known natural product BGCs located in plasmids.

Cyanobacterial plasmids have previously been shown to contain genes encoding RiPPs and are associated with the production of toxic and odorous compounds (Bolch et al., 1997; Wang et al., 2011). These replicons are also believed to play a major role in the distribution and evolution of toxin biosynthetic pathways in cyanobacteria (Dittmann et al., 2015). Plasmid-encoded natural product BGCs were manually curated in the present study and contained all the core genes for the biosynthetic pathways of the hepatotoxin microcystin, the antifungal hassallidin, and the odorous terpenoid geosmin (Kaebernick et al., 2000; Vestola et al., 2014; Suurnäkki et al., 2015). A biosynthetic pathway of the antiproliferative cytotoxin cryptophycin is located in a smaller plasmid of *Nostoc* sp. strain ATCC 53789 and is known to produce the natural product (Panda et al., 1998; Tippelt et al., 2020). Therefore, chemical analyses of the strains included in this study could determine that the biosynthetic pathways located in plasmids could be producing the compounds. Supporting this hypothesis, PPTs were also located in almost all analyzed cyanobacterial genomes.

PPTs have an important role in the biosynthesis of most analyzed classes of natural products in the present study (Hopwood, 1997; Marahiel et al., 1997). Previous mapping of PPTs in cyanobacteria indicated that only one copy occurred per genome (Copp and Neilan, 2006). Here, up to three different PPTs were found. Corroborating our results, other bacteria also contain multiple copies of these enzymes (Beld et al., 2014; Kim et al., 2018). Moreover, most of the predicted enzymes in the analyzed cyanobacterial genomes corresponded to the known lengths of PPTs (115–230 amino acids; Lambalot et al., 1996; Quadri et al., 1998). Two main families of PPTs are known, namely AcpS-type PPTs, which are involved in activating carrier proteins involved in primary metabolism, and Sfp-like PPTs as part of secondary metabolism pathways (Lambalot et al., 1996; Quadri et al., 1998). Sfp-like PPTs were the sole type found in cyanobacterial genomes (Copp and Neilan, 2006). However, *Acaryochloris marina* MBIC11017 possibly possesses a representative of an AcpS-like PPT in a plasmid. Consistent with our results, plasmids from other bacterial phyla have also been found to encode PPTs (Liu et al., 2005; De Lay and Cronan, 2008). This enzyme is in a cyanobacterial plasmid and may have originated from a horizontal gene transfer.

The replicon pCC7120α from *Nostoc* sp. PCC7120 is the only cyanobacterial plasmid that has been reported to be transmissible *in vivo* (Muro-Pastor et al., 1994). A previous study using automatic annotation found no homologs of T4SS in cyanobacteria and hypothesized that an unknown mechanism of conjugation could be present in these organisms (Smillie et al., 2010). Later studies investigated some of these possible mechanisms, such as integrative conjugative elements (ICE) and origin-of-transfer (*oriT*) sequences. ICEs are conjugative elements integrated into chromosomes (Guglielmini et al., 2011), whereas *oriT* are present in non-conjugative plasmids that can be mobilized by the relaxase in conjugative plasmids (O’Brien et al., 2015). Considering that ICEs and *oriT* are relatively less explored than the genes involved in conjugation, the mobility of the analyzed cyanobacterial plasmids was predicted exclusively on the genes for conjugation (Smillie et al., 2010).

We have provided evidence using manual curation that some plasmids in cyanobacteria may be mobile and contain natural product BGCs. These should be tested *in vivo* to expand the number of known conjugative plasmids from those organisms and to determine if the predicted cyanobacterial natural product BGCs are being transferred through conjugation. Toxic natural products produced by other bacteria, such as botulinum toxin from *Clostridium botulinum* and cereulide from *Bacillus cereus* were also found on plasmids (Ehling-Schulz et al., 2006; Carter et al., 2014). In the case of the botulinum toxin, horizontal gene transfer of BGC by large conjugative plasmids is also believed to be possible (Skarin and Segerman, 2011).

## Conclusion

The availability of complete genomes allowed mapping of many natural product BGCs in cyanobacterial plasmids. Manual curation identified the biosynthetic pathways of known toxins (microcystin), odorous metabolites (geosmin), protease inhibitors (anabaenopeptin, aeruginosin), and antimicrobial (ambiguine and hassalidin), and antitumor (cryptophycin) compounds. These biosynthetic pathways in plasmids included core genes necessary for biosynthesis. PPTs, a key enzyme for biosynthesis of many of these natural products, was found in most of the genomes. Therefore, we predict that these plasmid-based biosynthetic pathways are likely able to produce natural products. Moreover, the plasmid containing the geosmin BGC and others with cryptic natural product BGCs were predicted to be mobile. This is novel *in silico* evidence that plasmids are involved in the dissemination and evolution of diverse natural product biosynthetic pathways in cyanobacteria. If confirmed, the transmission of natural product BGCs among cyanobacteria by conjugation would present new biotechnological opportunities but also several risks. Cyanobacterial taxa which are not known to be problematic could acquire genes for toxin biosynthesis and cause economic losses, threats to public health, and damage to natural environments. Future research should investigate *in vivo* the conjugation of cyanobacterial plasmids and the potential transmission of BGCs among cyanobacteria. Likewise, chemical analyses of the strains included in the present study may lead to the discovery of novel natural products and chemical variants of known compounds.

## Data Availability Statement

The datasets presented in this study can be found in the Supplementary Material.

## Author Contributions

RP, DA, and DF conceptualized the study. RP, DA, and RC-B performed the analyses. RP wrote the manuscript. DF and KS were responsible for supervision. KS managed funding acquisition. All authors participated in reviewing and editing the manuscript and agreed to the published version of the manuscript.

## Conflict of Interest

The authors declare that the research was conducted in the absence of any commercial or financial relationships that could be construed as a potential conflict of interest.

## Publisher’s Note

All claims expressed in this article are solely those of the authors and do not necessarily represent those of their affiliated organizations, or those of the publisher, the editors and the reviewers. Any product that may be evaluated in this article, or claim that may be made by its manufacturer, is not guaranteed or endorsed by the publisher.

## Footnotes

1
https://www.r-project.org

2
https://inkscape.org/

3
http://tree.bio.ed.ac.uk/software/figtree/

## Supplementary Material

Supplementary Figure 1 Boxplot of the number of automatically annotated biosynthetic gene clusters identified in the cyanobacterial genera with more than one representative deposited in the NCBI GenBank (Clark et al., 2016). The genera are presented in increasing order of the average number of gene clusters (represented by “x”).

Supplementary Figure 2 Visualization of *Gloeothece verrucosa* PCC 7822 plasmid Cy782201. The position of the five automatically annotated biosynthetic gene clusters are indicated. Similarities with known BGCs calculated by antiSMASH v5.1.1 (Blin et al., 2019) are also indicated.

Supplementary Figure 3 Known natural products biosynthetic gene clusters (BGCs) and proteins involved in conjugation (MOB + MPF_c_) identified in cyanobacterial plasmids. The location and organization of BGCs in their respective plasmids are shown. See Supplementary Table 1 for information on gene annotation. The positions of manually annotated BGCs in the genomic maps are highlighted in blue or red.

Supplementary Figure 4 16S rRNA phylogeny of the 185 analyzed genomes **(A)** and representation of known BGCs found in plasmids (shown in bold) and chromosomes **(B–E)**. The posterior probability of clades is shown in the trees. NCBI GenBank (Clark et al., 2016) accession numbers are presented in parentheses. 16S rRNA genes of *Synechococcus elongatus* PCC 11801 and *Synechococcus elongatus* PCC 11802 were not available in publicly available genomes.

Supplementary Table 1 Manual curation of known biosynthetic gene clusters found in cyanobacterial plasmids. See “Materials and Methods” section for further information.

Supplementary Table 2 Annotation of 4-phosphopantetheinyl transferases in the analyzed cyanobacterial genomes. See the “Materials and Methods” section for further information.

Supplementary Table 3 Manual curation of genomic regions with relaxase, *virB4*, and *virD4* genes. Previous studies characterized the mobility of plasmids (Smillie et al., 2010), MOB_v_ (Garcillán-Barcia et al., 2009), and MPF_c_ (Guglielmini et al., 2011).

Supplementary Table 4 Predicted mobility of cyanobacterial plasmids. See the “Materials and Methods” section for further information.

Supplementary Table 5 List of public genomes deposited in the NCBI GenBank (Clark et al., 2016) used in the present work and the results of automatic annotation performed with antiSMASH v5.1 (Blin et al., 2019). See the “Materials and Methods” section and Supplementary Table 1 for information on the manually curated genes.
