# Supplementary figures and images for: Mining of Cyanobacterial Genomes Indicates Natural Product Biosynthetic Gene Clusters Located in Conjugative Plasmids
Source: Front Microbiol. 2021 Nov 4;12:684565. doi: 10.3389/fmicb.2021.684565 (PMC8600333; doi:10.3389/fmicb.2021.684565)

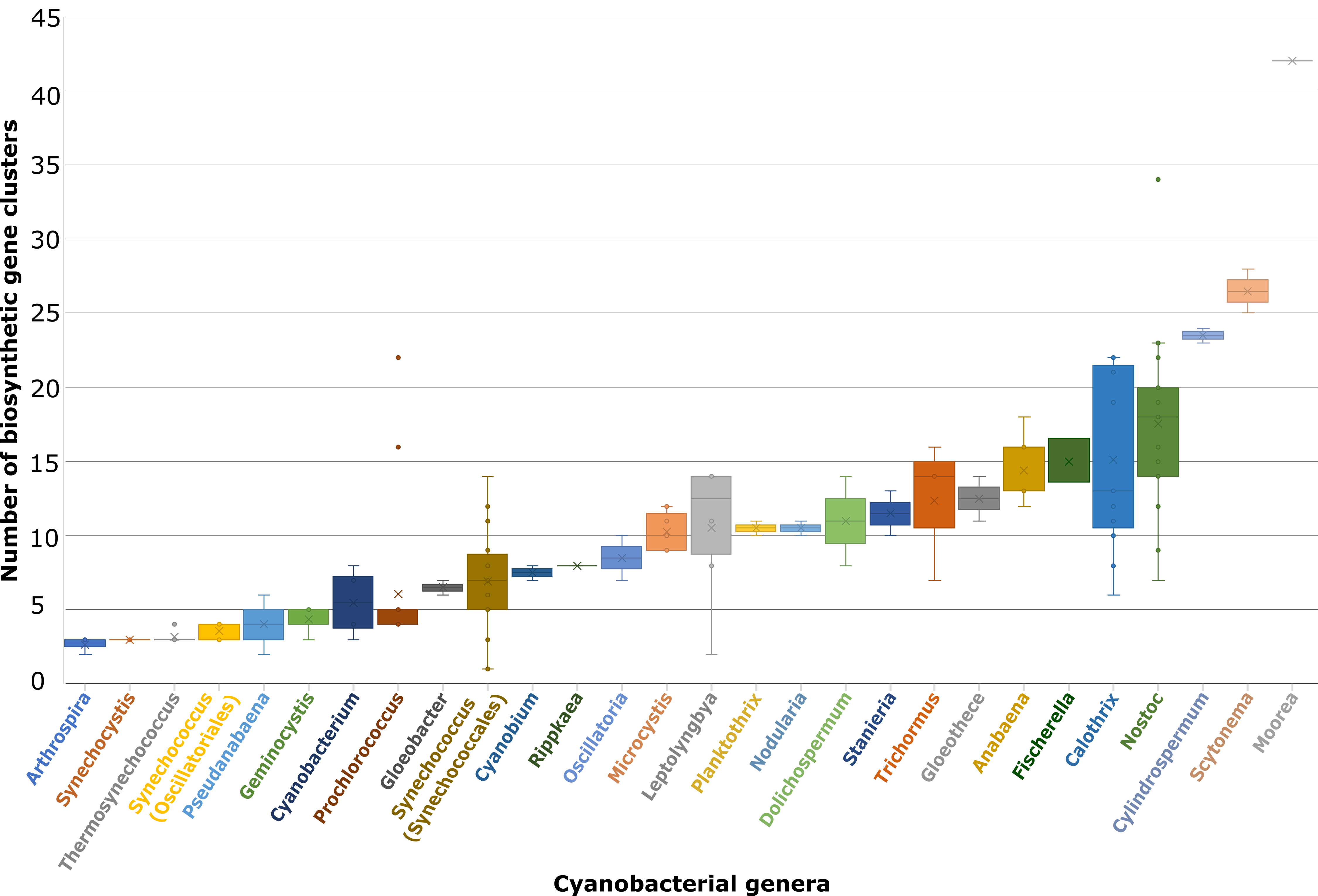

Supplement: Supplementary Figure 1 — Boxplot of the number of automatically annotated biosynthetic gene clusters identified in the cyanobacterial genera with more than one representative deposited in the NCBI GenBank (Clark et al., 2016). The genera are presented in increasing order of the average number of gene clusters (represented by “x”). [file Image_1.TIFF]

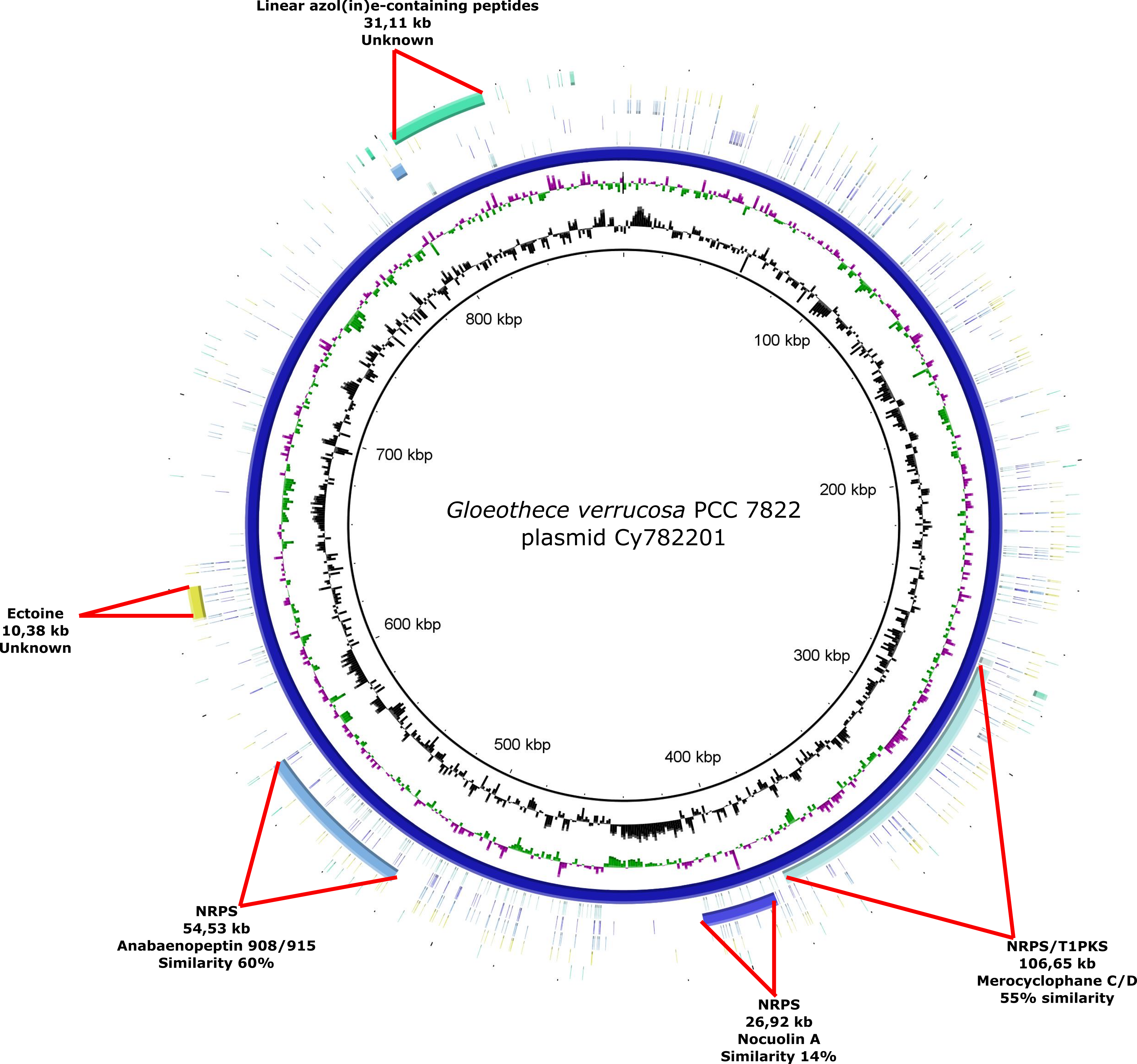

Supplement: Supplementary Figure 2 — Visualization of Gloeothece verrucosa PCC 7822 plasmid Cy782201. The position of the five automatically annotated biosynthetic gene clusters are indicated. Similarities with known BGCs calculated by antiSMASH v5.1.1 (Blin et al., 2019) are also indicated. [file Image_2.TIFF]

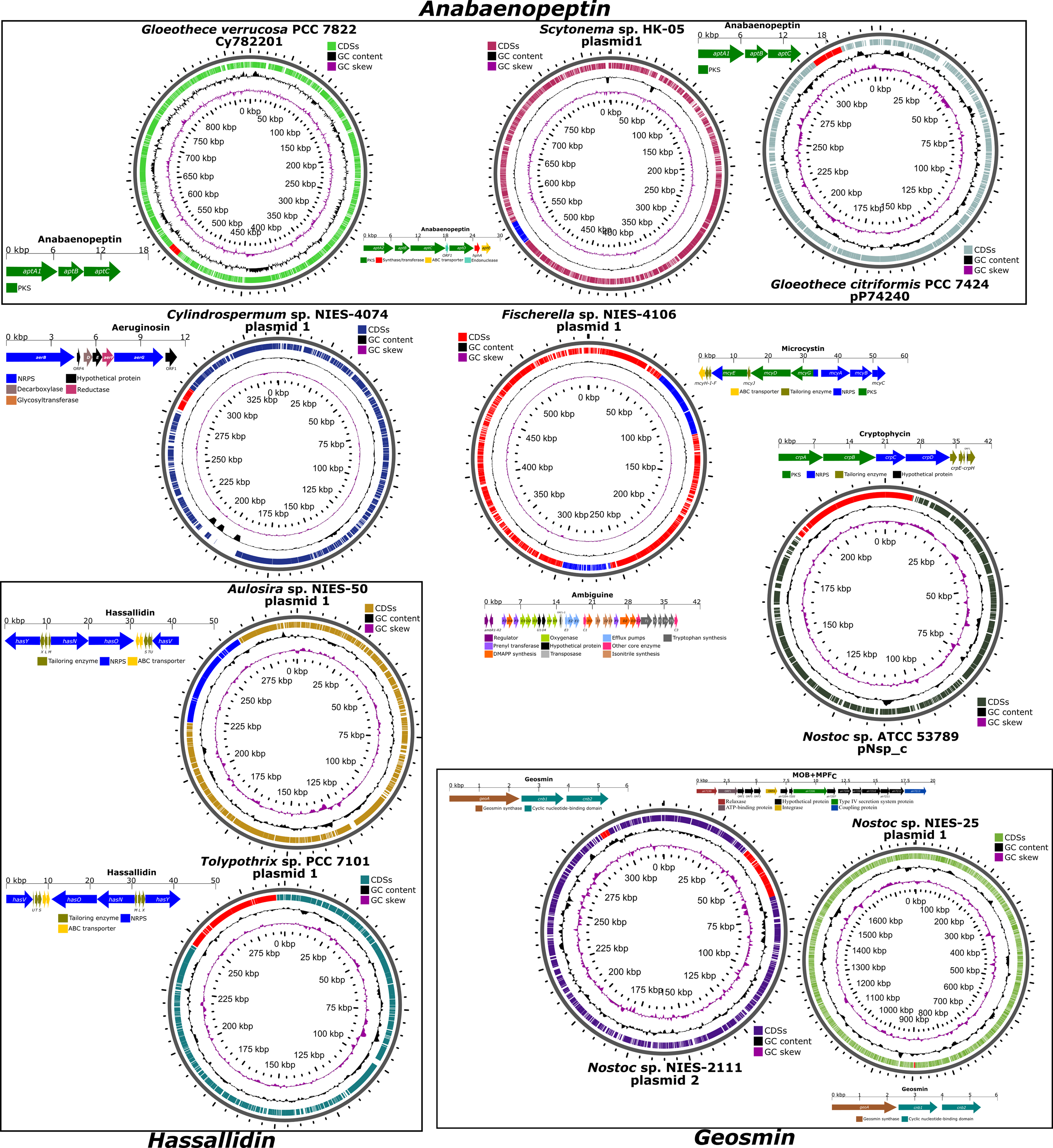

Supplement: Supplementary Figure 3 — Known natural products biosynthetic gene clusters (BGCs) and proteins involved in conjugation (MOB + MPFc) identified in cyanobacterial plasmids. The location and organization of BGCs in their respective plasmids are shown. See Supplementary Table 1 for information on gene annotation. The positions of manually annotated BGCs in the genomic maps are highlighted in blue or red. [file Image_3.TIFF]

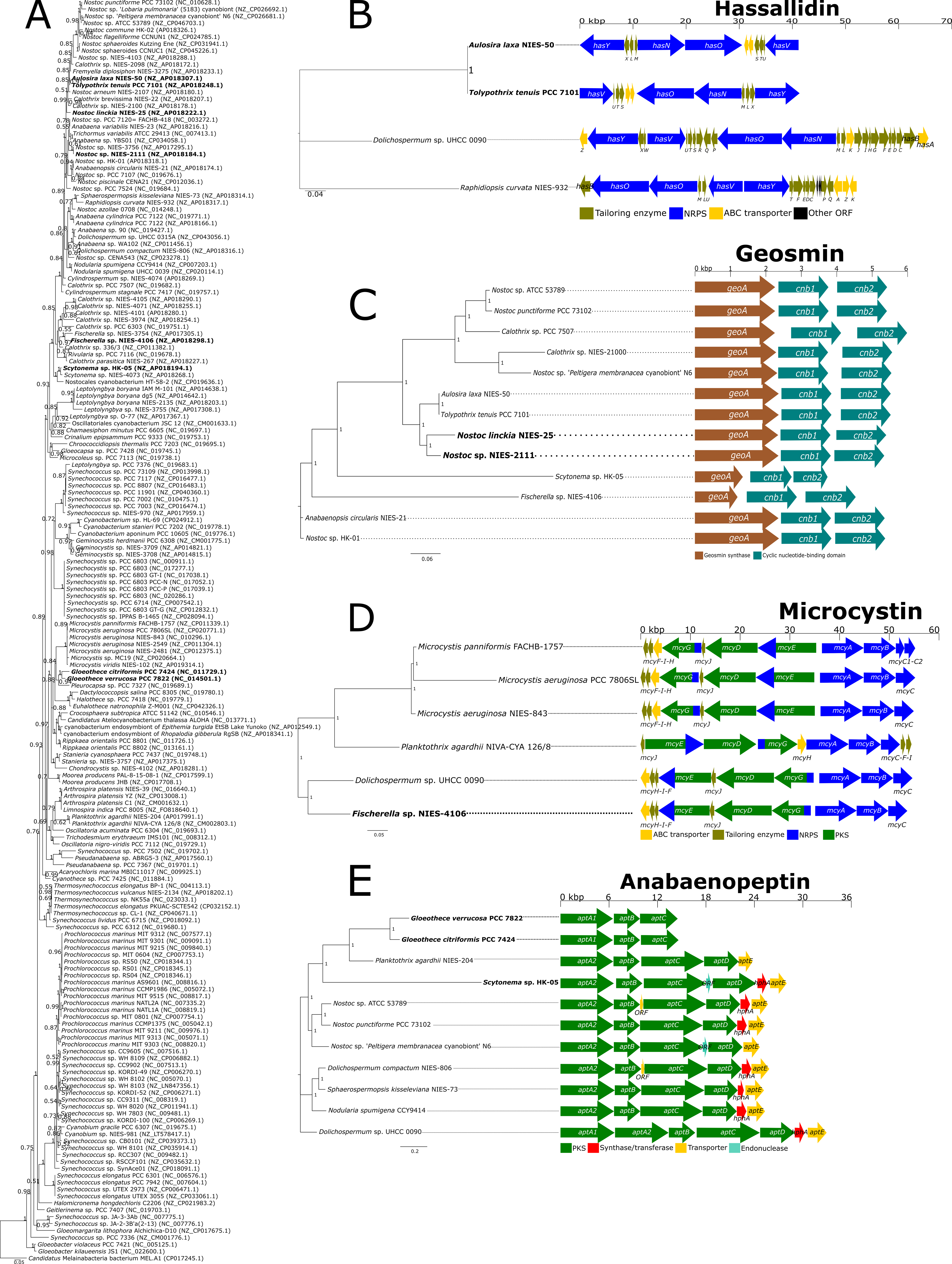

Supplement: Supplementary Figure 4 — 16S rRNA phylogeny of the 185 analyzed genomes (A) and representation of known BGCs found in plasmids (shown in bold) and chromosomes (B–E). The posterior probability of clades is shown in the trees. NCBI GenBank (Clark et al., 2016) accession numbers are presented in parentheses. 16S rRNA genes of Synechococcus elongatus PCC 11801 and Synechococcus elongatus PCC 11802 were not available in publicly available genomes. [file Image_4.TIFF]
